# Managing Prolactinomas during Pregnancy

**DOI:** 10.3389/fendo.2015.00085

**Published:** 2015-05-26

**Authors:** Mussa Hussain Almalki, Saad Alzahrani, Fahad Alshahrani, Safia Alsherbeni, Ohoud Almoharib, Naji Aljohani, Abdurahman Almagamsi

**Affiliations:** ^1^Obesity, Endocrine, and Metabolism Center, King Fahad Medical City, Riyadh, Saudi Arabia; ^2^College of Medicine, King Fahad Medical City, King Saud bin Abdulaziz University for Health Science, Riyadh, Saudi Arabia; ^3^College of Medicine, King Abdulaziz Medical City, King Saud bin Abdulaziz University for Health Science, Riyadh, Saudi Arabia

**Keywords:** prolactin, pituitary tumor, dopamine agonist, pregnancy and outcome

## Abstract

Prolactinomas are the most prevalent functional benign pituitary tumors due to a pituitary micro- or macroadenoma. The majority of patients presents with infertility and gonadal dysfunction. A dopamine agonist (DA) (bromocriptine or cabergoline) is the treatment of choice that can normalize prolactin levels, reduce tumor size, and restore ovulation and fertility. Cabergoline generally preferred over bromocriptine because of its higher efficacy and tolerability. Managing prolactinomas during pregnancy may be challenging. During pregnancy, the pituitary gland undergoes global hyperplasia due to a progressive increase in serum estrogens level that may lead to increase of the tumor volume with potential mass effect and visual loss. The risk of tumor enlargement may occur in 3% of those with microadenomas, 32% in those with macroadenomas that were not previously operated on, and 4.8% of those with macroadenomas with prior ablative treatment. Though both drugs appear to be safe during pregnancy, the data on fetal exposure to DAs during pregnancy have been reported with bromocriptine far exceeds that of cabergoline with no association of increased risk of pregnancy loss and premature delivery. It is advisable to stop the use of DAs immediately once pregnancy is confirmed, except in the case of women with invasive macroprolactinomas or pressure symptoms. This review outlines the therapeutic approach to prolactinoma during pregnancy, with emphasis on the safety of available DA therapy.

## Introduction

Prolactinomas are adenomas arising from lactotroph cells in the pituitary gland that secrete prolactin, and are considered the most frequently diagnosed functioning pituitary tumor type, accounting for about 40% of all pituitary adenomas (1). Prolactin production and release is mediated via tonic inhibition by dopamine secreted by the hypothalamus. The breast is the main target tissue for prolactin, but prolactin receptors have been found in several tissues, including the liver, ovary, testis, and prostate (2). The primary action of prolactin is the initiation and maintenance of lactation, but it can act as a growth factor, neurotransmitter, or immunoregulator via autocrine or paracrine mechanisms (3).

Prolactinomas are one of the causes of hyperprolactinemia, a condition in which 90% are intersellar adenomas and 10% are macroadenomas (≥10 mm) (4). Prolactinomas occur with a prevalence of 60–100 cases per million (1). It is more common in women, particularly during the reproductive period. The highest incidence rate was found in women between 25 and 34 years of age: 23.9/100,000 person-years (4). In women, prolactinomas are usually microadenomas (<10 mm) presenting with high-prolactin levels, which leads to amenorrhea, galactorrhea, and infertility. Men with prolactinomas frequently present with symptoms of mass effects, such as headache and visual loss, and sometimes hypogonadism and infertility. Bone loss may occur as a long-term consequence of hyperprolactinemia in both men and women.

Dopamine agonists (DAs) have been increasingly used as a treatment of choice for prolactinomas as it normalizes prolactin levels and leads to tumor shrinkage. Surgery currently being reserved for resistance or intolerance to DA, while radiotherapy used only in cases of surgical failure or resistance to DA.

During pregnancy, the pituitary gland undergoes global hyperplasia due to a progressive increase in serum estrogens level that may lead to increase of the tumor volume with potential mass effect and visual loss, which poses significant challenge to endocrinologists.

## Prolactinoma during Pregnancy

Tumor cells in patients with prolactinomas express estrogen receptors; as a result of the increased estrogen level during pregnancy (5), there can be a substantial increase in the volume of the prolactinoma, with a progressive increase in serum prolactin due to lactotroph cell hyperplasia (6). The main concern is possible tumor enlargement during pregnancy. The risk of tumor enlargement during pregnancy is found to depend on tumor size. Data in the literature indicate that although tumor enlargement is only 3% for microprolactinomas, it is as high as 32% for macroprolactinomas that were not previously operated on (7).

A magnetic resonance imaging (MRI) should be done before conception to document tumor size and to serve as a baseline for comparison with MRIs done during pregnancy. Furthermore, MRI is helpful in distinguishing between hemorrhage into a tumor versus simple tumor enlargement during pregnancy (8).

Although prolactinomas have been found to be amenable to medical treatment during pregnancy, especially with DAs such as bromocriptine, not enough safety data are available to recommend the routine use of these drugs during pregnancy. The endocrine society clinical practice guidelines for the management and treatment of prolactinomas recommend the discontinuation of DAs shortly after confirmation of pregnancy, with the exception of women with invasive macroprolactinomas (5).

Bromocriptine is an ergoline derivative, a dopamine D2 receptor agonist with agonist and antagonistic properties on D1 receptors. It is generally required to multiple dosing throughout the day because of its short half life (9–11). In women taking bromocriptine during early pregnancy, the incidence of abortions, ectopic pregnancies, or congenital malformations is no higher than that in the general population (12). In a study of 2,587 pregnant women, out of which 2,437 were to increase the risk of spontaneous abortion, congenital abnormalities, or multiple pregnancies, and did not affect post-natal development (13). In other studies, among 6,329 patients treated with bromocriptine during early pregnancy, the risk of spontaneous abortions was 9.9%, which was not higher than that in the general population (10.9%) (7, 14). Moreover, long-term follow-up to 9 years of children born from mothers taking bromocriptine did not cause detrimental effects on fetal outcomes in term of physical development as well as no psychomotor developmental abnormality reported to 5.5 years (1–20 years) follow-up (15).

Furthermore, optimal outcome was found with continuous use of bromocriptine throughout pregnancy in around 100 cases (16).

Cabergoline is an ergoline derivative DA with higher affinity and selectivity for D2 dopamine receptors. It has long duration of action allowing administration once or twice weekly with better tolerability and patient compliance (17–20). Moreover, pregnancy rate is higher with cabergoline in infertile women with prolactinoma than with bromocriptine (21).

The same results have been reported in women who were on cabergoline before and during pregnancy (22, 23). In one report, over 800 such pregnancies have been described (24) (of which approximately 350 were exposed during the first weeks of pregnancy), with no significant difference in the frequency of spontaneous abortion, premature delivery, multiple pregnancy, or neonatal malformations (25, 26).

In retrospective study on 103 pregnancies in 90 women with hyperprolactinemia and the follow-up of the 61 children, no significant abnormalities related neither to cabergoline doses nor to the time of exposure (27). Data of 12 years follow-up in the children born from mother treated with cabergoline showed no influence on their post-natal development such as physical problems or of psychomotor retardation (28). In consistent with previous studies, finding from meta-analysis showed no significant increase in the risk of miscarriages or fetal malformation with DA used (29).

Quinagolide is a non-ergot DA, with selective dopamine-2 receptor agonist and long-lasting prolactin lowering effect. It generally taken once daily and has better tolerability and convenient dosing schedule than bromocriptine (13, 30, 31). It has limited safety compared with bromocriptine. In a review of 176 pregnancies in which quinagolide was used (average of 37 days), spontaneous abortion occurred in 14%, 1 had premature delivery, and 1 had ectopic gestation (13). In another small study of nine pregnancies, there were two cases treated with two quinagolide and had no complications (31).

Over 200 pregnancies have been recorded in women taking quinagolide, and no apparent adverse effects on pregnancy or fetal development have been detected (32).

Hence, every woman with prolactinomas should be made aware of the natural history of prolactinomas and should discuss her plans to conceive with her physician, in order to be carefully evaluated prior to becoming pregnant.

**Figure 1:**
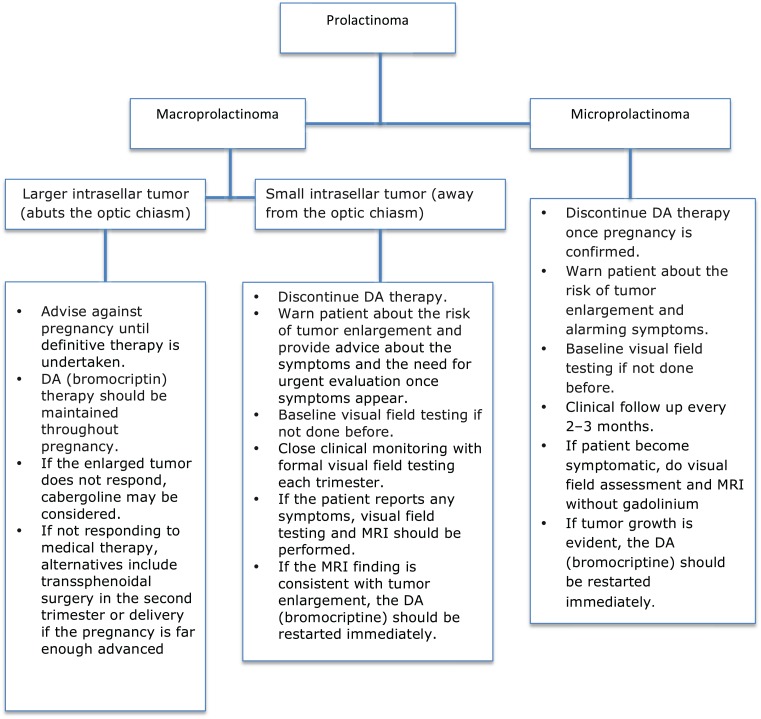
**Approach to managing prolactinomas during pregnancy**.

The determination of the appropriate treatment option is an individual choice depending mainly on tumor size. The proposed therapeutic approach is shown in Figure 1 (33). The outcomes of prolactinomas after pregnancy have been extensively discussed, with variable results. A recent study showed a prolactin normalization rate of more than 40% without medical treatment, for a median follow-up of 22 months after delivery and cessation of lactation (34).

## Managing Microprolactinomas during Pregnancy

Microprolactinomas in non-pregnant women tend to follow a benign course. The risk of significant asymptomatic tumor growth during pregnancy is 4.5% (35), and that of symptomatic tumor growth is <2% (36). The risk of the development of new neurological sequelae (headaches, optic nerve compression, etc.) ranges from 1.6 to 5.5% (37). Prolactin tends to increase during pregnancy; therefore, it does not reliably reflect an increase in tumor size and is not useful for clinical assessment.

In the context of the very low risk of microprolactinoma enlargement during pregnancy, there is considerable evidence supporting the discontinuation of DA treatment once pregnancy is confirmed. The patient should be told that the risk of enlargement of the adenoma during pregnancy is very small, and medical treatment will likely be effective if symptoms do occur.

The patient should be advised to report for urgent assessment in case of unusual symptoms such as severe headache or visual disturbance, to rule out the possibility of tumor enlargement. Serial prolactin determinations are not necessary due to the high variability of prolactin during pregnancy (38).

The patient should undergo baseline formal visual field testing at the time of diagnosis and should be followed clinically every 2–3 months during pregnancy (33), but serial MRI examinations or visual field testing during pregnancy is not required. However, there is no data documenting harm to the fetus from either MRI scans or gadolinium (8).

In case the patient becomes symptomatic with visual disturbance or progressive headaches, an MRI without gadolinium (not a CT) should be performed to assess changes in tumor size (39).

If substantial tumor growth is evident, the DA bromocriptine should be restarted immediately (36), because it is the first drug of choice in these cases.

For women who remain symptom-free throughout pregnancy, serum prolactin should be measured 2 months after delivery or cessation of nursing, and if it is similar to the pretreatment value, the drug should be restarted (40). Similarly, for women wishing to breastfeed, an MRI scan should be done to ensure the stability of the tumor within 4–6 weeks of delivery (37), as DAs will decrease serum PRL levels, subsequently impairing lactation.

## Managing Macroprolactinomas during Pregnancy

In women, macroprolactinomas occur less frequently than microprolactinomas. In case of macroprolactinoma, symptomatic tumor enlargement occurs in 20–30% of cases (41). It has been reported that the risk of clinically significant tumor enlargement falls from over 30 to <5%, if the patient is treated with radiation or surgery before pregnancy (25).

Pregnant women with large tumors and those with extrasellar extension who have stopped bromocriptine are at risk for tumor growth, and formal visual field testing should be done in each trimester. Just like in microprolactinomas, it is not necessary to measure serum prolactin levels throughout pregnancy, because levels do not uniformly increase during gestation and do not correlate with tumor enlargement (8, 25). Furthermore, in these cases, the treatment should be individualized, as data comparing various management strategies are lacking.

The patient should be informed about the relatively higher risk of tumor enlargement, the need for normalization of prolactin, and the importance of treatment before conception.

In the case of intrasellar and small macroprolactinomas that do not abut the optic chiasm, DAs should be discontinued when pregnancy is confirmed.

The patient should be advised about the symptoms and the need for urgent evaluation once they appear. Close clinical monitoring should be undertaken with formal visual field testing during each trimester. If the patient reports headache or a change in vision, an MRI should be performed. If the MRI finding is consistent with tumor enlargement, the woman should be retreated with a DA (5).

Bromocriptine is considered as the first drug of choice, and it usually decreases the size of the adenoma and eliminates symptoms (42). Cabergoline may be considered if the adenoma does not respond to bromocriptine (43).

If the enlarged tumor does not respond to cabergoline therapy, alternatives include transsphenoidal surgery in the second trimester, or delivery if the pregnancy is advanced enough (16, 25, 41).

Patients with large macroprolactinomas and those with extrasellar extension are strongly discouraged from conceiving until definitive therapy is undertaken. Therapy with surgical debulking may be considered prior to pregnancy, because surgery or radiation has been shown to decrease the risks of symptomatic tumor enlargement (25), but there is high risk of hypopituitarism in these cases. However, debulking surgery is a less preferable option, since medical therapy during pregnancy is probably less harmful than surgery (41). Women with prolactinomas that are resistant to DAs are usually infertile. Therefore, pregnancy is unexpected in these cases, unless they undergo ovarian stimulation with gonadotropins or GnRH. Moreover, pregnancy is not recommended in women with drug resistant large macroprolactinomas and they should not conceive even if the tumor is intrasellar, until the size is reduced by transsphenoidal surgery.

Surgery is an option in cases with no tumor reduction during medical treatment with DA, or in those who developed tumor growth in a previous pregnancy (44).

## Impact of Pregnancy and Breastfeeding on Prolactin Levels, Tumor Volume, and Remission Rate

The current literature demonstrates that pregnancy induces remission of hyperprolactinemia in two-thirds of women after discontinuation of DA. In one study, pregnancy has been found to induce remission in 76% of non-tumoral hyperprolactinemia (NTHP), 70% in microprolactinomas, and 64% in macroprolactinomas with higher recurrence rate among patients with macroprolactinomas and those with microprolactinomas with visible tumor on MRI at the time of treatment withdrawal (45).

In recent study, complete remission was found in 100% with NTHP, 66% of patients with microprolactinomas, and 70% with macroprolactinomas (46). Underlying mechanisms are uncertain but have generally been attributed to the autoinfarction of the tumor (46).

On the other hand, there is no data to suggest breastfeeding is associated with an increased prolactin production or risk of tumor enlargement (28, 46). Thus, women could breastfeed normally and restart DA after cessation of lactation.

## Conclusion

Managing prolactinoma during pregnancy is challenging. Clinical trials highlighting the outcomes of medical therapy versus other therapies are scarce; therefore, the patient is treated on an individual basis. The outcome of microadenoma is excellent, allowing patients to safely discontinue DAs with close clinical monitoring; on the other hand, macroadenoma needs to be managed before conception, as the risk of tumor enlargement is relatively high.

## Conflict of Interest Statement

The authors declare that the research was conducted in the absence of any commercial or financial relationships that could be construed as a potential conflict of interest.
